# Magnetic Domain Patterns in Bilayered Ribbons Studied by Magnetic Force Microscopy and Magneto-Optical Kerr Microscopy

**DOI:** 10.1155/2018/8308460

**Published:** 2018-03-26

**Authors:** Jana Trojková, Ondřej Životský, Aleš Hendrych, Dmitry Markov, Klára Drobíková

**Affiliations:** ^1^Department of Physics, VŠB-Technical University of Ostrava, 17 Listopadu 15/2172, Poruba, 708 33 Ostrava, Czech Republic; ^2^Boris Yeltsin Ural Federal University, Ekaterinburg 620002, Russia; ^3^Varroc Lighting Systems, s.r.o., Suvorovova 195, 742 42 Šenov u Nového Jičína, Czech Republic; ^4^IT4Innovations Centre of Excellence, VŠB-Technical University of Ostrava, 17 Listopadu 15/2172, Poruba, 708 33 Ostrava, Czech Republic; ^5^Nanotechnology Centre, VŠB-Technical University of Ostrava, 17 Listopadu 15/2172, Poruba, 708 33 Ostrava, Czech Republic

## Abstract

The magnetic domain patterns of amorphous bilayered FeSiB/FeNbSiB and FeNbCuSiB/CoSiB ribbons are observed and analysed using the magneto-optical Kerr microscopy (MOKM) and magnetic force microscopy (MFM). Both microscopic techniques are highly sensitive to the sample surface; possibility of Kerr microscopy to visualize the domains separately in both layers is achieved by focusing the laser spot on the ribbon cross section. Wide curved domains as well as fine fingerprint domains were detected at the surface of ribbons due to presence of local stresses coming from the preparation process. With respect to high lateral resolution of MFM and its out-of-plane magnetization sensitivity, the perpendicularly magnetized crossed stripe domain patterns can be selected as well. Coiling of the ribbons on the half-round-end sample holder is often used to induce and control the magnetic anisotropy of these alloys. Changes in the magnetic domain structure at the outer-coiled surface and its dependence on the sign of magnetostriction coefficient are discussed in detail. Finally, the MFM images in the presence of external in-plane magnetic field up to ±40 kA/m are shown.

## 1. Introduction

The amorphous and/or nanocrystalline alloys are deeply examined by many research teams world-wide due to their excellent soft magnetic properties [1, 2]. They are produced in many forms and geometries (ribbons, wires, and thin films) by various techniques [1, 3, 4]. Besides other fabrication methods suitable for production of soft magnetic materials the planar flow casting (PFC) is referred to as most utilizable [5].

Recently, the innovations of PFC technology connected with the integration of double-nozzle allow the preparation of bilayered (BL) and/or multilayered (ML) functional materials. They are used mainly in sensor applications, like deflection sensors [6] and displacement sensors [7] and also as ferromagnetic shape-memory alloys [8] or as alloys with enhanced magnetocaloric [9] and GMI effect [10]. The initial production of monolithic BL system started back in the 1990s, where two compositions of FeNiB/CoFeCrSiB were put together [11]. Promising step towards the development of such structures resulted in the inhomogeneous properties of mentioned layers mainly due to the fact that both compositions were separated in two crucibles during the injection on rotating wheel. Consequent efforts led to the production of BL ribbons involving one crucible with separated chambers for production of bilayered ribbons, where two melts are cast almost at the same time. Since that time, the preparation process has been significantly improved [6] leading to better homogeneity of the layers and interface [12]. Particular applications of these materials are closely related to the magnetic anisotropy originated in the bulk and on the surface during the ribbon preparation process [13]. However, the changes of magnetic anisotropy in these soft magnetic materials are reflected by the magnetic domain patterns.

Nowadays, the magnetic force microscopy (MFM) is advanced well established surface-sensitive technique for magnetic domain observations in a variety of magnetic materials (e.g., recording media [14], particles [15], nanocomposites [16, 17], amorphous and/or nanocrystalline alloys [18–20], and thin films [21]). It is considered as easy available micromagnetic method with sufficient resolution; on the other hand, quantitative expertise of MFM images remains debatable and still very challenging. Soft magnetic materials studied by MFM are very sensitive to the perturbation effects of the tip (thin ferromagnetic films) or sample stray fields and their mutual changes during measuring process. As a consequence the domain structures are occasionally hard to interpret. Anyway, there is an optical technique based on magneto-optical Kerr effect (MOKE) suitable for detection of surface magnetic properties in these alloys. Surface magnetic anisotropy and depth sensitivity are often investigated by measuring the MOKE hysteresis loops [22, 23]. Magnetic domains from the near-surface region are observed using the magneto-optical Kerr microscopy (MOKM) based on light polarization and its change after reflection from the sample surface [24]. The resolution achieved by MOKM compared to the MFM is lower and strictly limited by the resolution of an optical element (objective). Despite this the MOKM offers fast measurements directly sensitive to the sample magnetization with sufficient contrast of magnetic images and therefore could serve as a proper tool for interpreting MFM response. The combination of both techniques has been successfully presented, for example, on Co and NdFeB crystals [25], where the force sensor was integrated into the objective revolver of an optical polarization microscope, on Fe-Ga bulk alloys [26], or on a single iron crystal [27].

The aim of the paper is complex observation and analysis of magnetic domains in bilayered FeSiB/FeNbSiB and FeNbCuSiB/CoSiB amorphous ribbons. We benefit from high resolution of MFM setup compared with MOKM flexibility and direct interpretation of domain images. MOKM technique is used to detect the induced magnetic anisotropy that is changing during the ribbon fixing on the half-round-end sample holder. Possibility of obtaining magneto-optical contrast at the cross section of both bilayered samples is presented. MFM domain patterns without and with the presence of external magnetic field are also deeply investigated.

## 2. Materials and Methods

As-cast, 36 *μ*m thick and 8 mm wide, amorphous bilayered Fe_77.5_Si_7.5_B_15_/Fe_74.5_Nb_3_Si_13.5_B_9_ (BL-FF) and Fe_73.5_Nb_3_Cu_1_Si_13.5_B_9_/Co_72.5_Si_12.5_B_15_ (BL-FC) ribbons were prepared by PFC technique using crucible divided into two chambers [6]. During the preparation process FeSiB and FeNbCuSiB layers of the sample were in contact with surrounding atmosphere (air side), while the opposite FeNbSiB and CoSiB layers were in contact with rotating wheel (wheel side). As confirmed by the X-ray diffraction (XRD) the ribbons are fully amorphous [13]. Basic structural and magnetic parameters of ribbons are summarized in Table 1.

**Figure 1:**
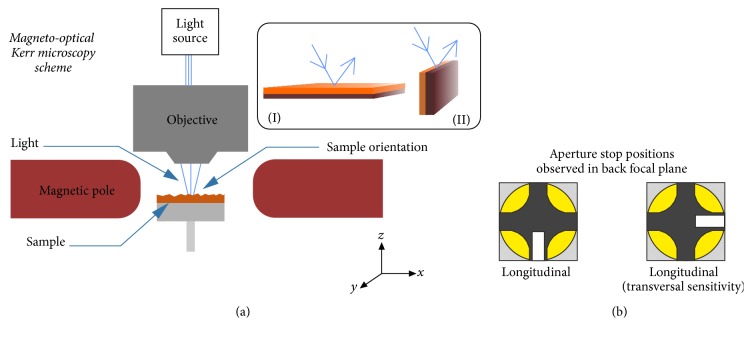
(a) Schematic description of magneto-optical Kerr microscope. Subplots (I) and (II) correspond to the surface and cross-sectional orientation of the samples with respect to the incoming light. In both cases the external magnetic field was applied along the *x*-axis of the coordinate system. (b) Sensitivity to in-plane magnetization components was ensured by the stop positions of aperture diaphragm.

Magnetic domains were investigated by two surface-sensitive techniques. Magneto-optical Kerr microscopy (MOKM) consists of specially designed polarization microscope for direct observation of magnetic domains; see schematic description in Figure 1. The white light from the Xe lamp passes through the system of optical elements composed of aperture diaphragm, polarizer, and polarization objective and incidents of the sample surface. Reflected light goes through the analyser almost crossed with the polarizer. Such arrangement is necessary for optimal domain contrast that is obtained by subtracting two images. Firstly we apply the magnetic field *H*_*s*_ necessary to saturate the sample and the surface image is stored as a reference. Then the value of magnetic field is gradually decreased and we observe the difference between the actual image at applied magnetic field *H* < *H*_*s*_ and reference. In most cases the MOKM domains were investigated in remnant state, that is, after switching off the magnetic field (*H* = 0). Sensitivity to individual magnetization components can be adjusted using the aperture diaphragm. Opening and closing of the diaphragm enable illuminating different areas of the conoscopic image, “Maltese cross,” occurring in the microscope back focal plane. As seen in Figure 1 at all MOKM experiments the light was screened to incident the edge part of conoscopic image and in this way the sensitivity to magnetization components lying in the ribbon plane (longitudinal, longitudinal with transversal sensitivity) was obtained. However, it is well known that out-of-plane (polar) magnetization component is also present due to oblique angle of light incidence. Subplots (I) and (II) show the orientation of the BL ribbon while measured with respect to the incoming light. To visualize the magnetic patterns at the cross section of the BL ribbon, the special sample holder was fabricated [12]. Vertical position of the sample is ensured by plastic clamp mounted into the acrylic case. The surface of the cross section of the BL ribbon was treated by grinding wheel with fine grain sizes and additionally polished for 1 hour using the Vibromet machine.

MOKM magnetic domain investigation was supplemented by atomic/magnetic force microscopy (AFM/MFM) measurements with and/or without external magnetic field. The AFM/MFM experiments were carried out in air at room temperature with the scanning probe microscopy (SPM) platform (Ntegra Prima, NT-MDT, Russia) using the Co-Cr coated cantilevers (see Table 2) in semicontact (tapping-lift) mode. The tips were magnetized perpendicularly to the sample surface and MFM senses the vertical component of the derivative of force between the sample and the tip. The coercivity of the tips is up to 16 kA/m. Firstly, the topography of specimen is obtained, and then the magnetic contrast is achieved by lifting the probe into the distance of 250 nm above the surface. All images have been collected both at remnant state and/or as a function of external magnetic field.

**Figure 2:**
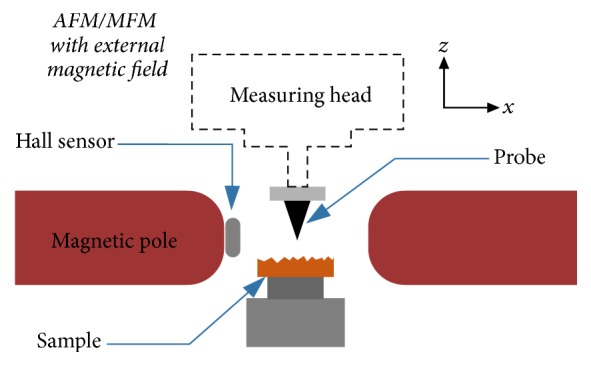
Schematic draft of AFM/MFM setup specially designed for measurements in external magnetic field generated along the *x*-axis.

The AFM/MFM setup takes advantage from longitudinal magnetic field generator (electromagnetic coil) that is able to create the magnetic field along the sample surface up to 80 kA/m (see Figure 2). To suppress the influence of metal parts on probe position while operated in magnetic field, the measuring head and exchangeable mount are made from nonmagnetic materials.

## 3. Results and Discussion

### 3.1. Magneto-Optical Kerr Microscopy

**Figure 3:**
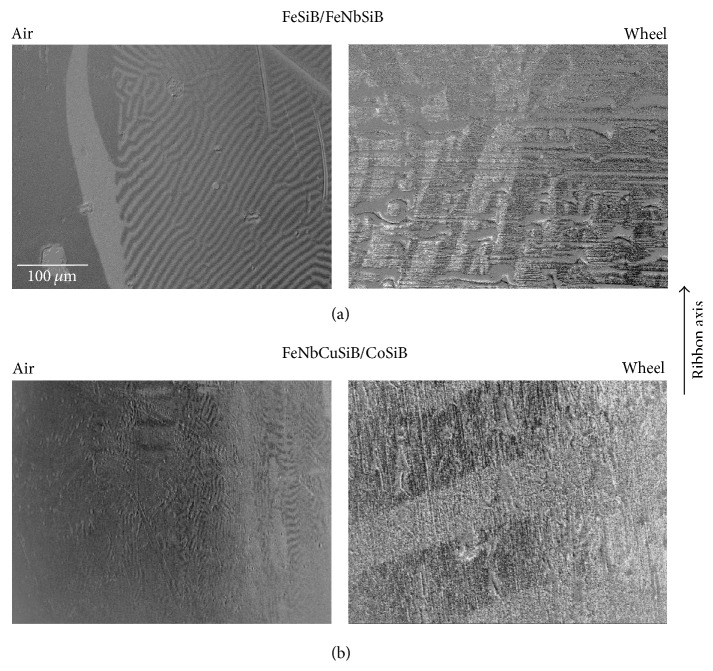
Surface magnetic domains obtained by MOKM at BL-FF (a) and BL-FC (b) ribbons in remnant state.

The surface domains of BL-FF and BL-FC samples measured by MOKM are given in Figure 3. As it is typical for amorphous ribbons we can distinguish two types of domain pattern. The origin of each is closely related to the preparation process leading to the introduction of local stresses. Fingerprint-like domains observed at air sides of both samples are understood as surface closure domains based on compressive tension. Their occurrence indicates the presence of out-of-plane magnetization component and local magnetic anisotropy perpendicular to the ribbon surface deeply in the bulk. In the near-surface region (a few tens of nm) the Kerr microscopy is sensitive mainly to their in-plane magnetization component. Presence of weak out-of-plane component can be easily verified at normal light incidence configuration, when the MO contrast practically diminishes. Contrary the wide curved domains originate as a consequence of tensile stress and follow the in-plane easy magnetization axis. Due to higher roughness on the wheel side of the samples (see Table 1), where the magnetic domains are overlapped by irregularities and structural defects coming from preparation, it is more complicated to visualize the magnetic pattern. There are no fingerprint domains; there is only the glimpse of wide domains that traces the direction of magnetization within the ribbon plane.

Generally we can say that each ribbon place will have its own unique magnetic domain structure reflecting on the one hand stresses coming from preparation process and on the other hand postpreparation treatment. Differences between the ribbons and their sides are visible from Figure 3. Due to preparation process the investigated BL-FF and BL-FC ribbons exhibit low coiling either with the air or wheel side out. Their uncoiling on the planar sample holder used for magnetic domain observations and the sign of magnetostriction coefficient of corresponding ribbon layer (see Table 1) are responsible for random fluctuations of the easy magnetization axis on the ribbon surface. Due to mentioned factors (i) the wide stripe domains have directions close to the ribbon axis on both sides of BL-FF ribbon, while on the wheel side of BL-FC sample they are rather perpendicular to it, and (ii) fingerprint-like domains corresponding to the prevailing planar compressive stress are visible partly on the BL-FF air surface and almost everywhere on the BL-FC air surface.

**Figure 4:**
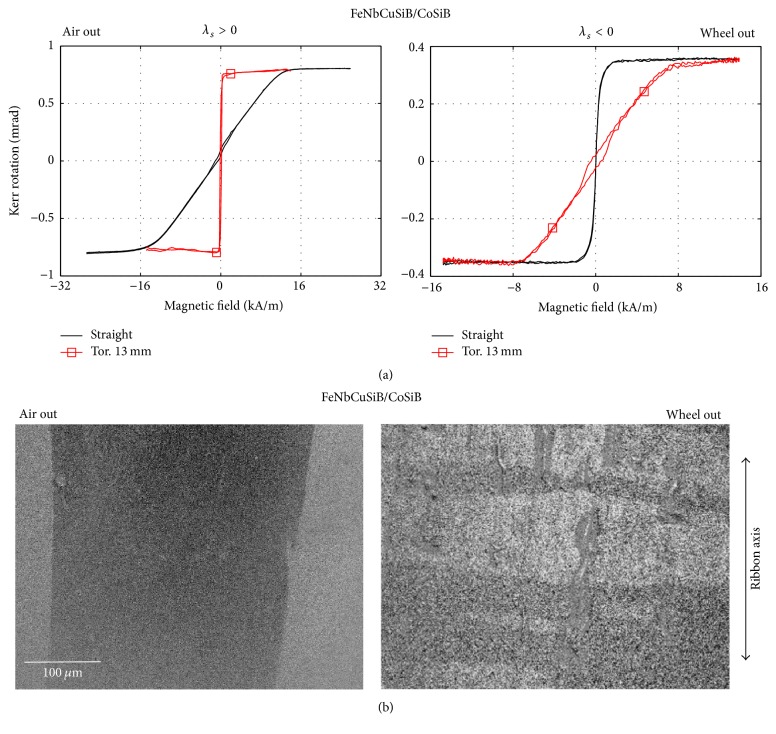
Magneto-optical hysteresis loops (a) and domain patterns (b) of the coiled BL-FC ribbon (toroid, 13 mm in diameter). Left and right subplot correspond to the coiling with the air and wheel side out, respectively. The loops and domains are measured from the outer sides.

Figure 4 shows the possibility of controlling the induced magnetic anisotropy due to coiling of the ribbons into the half-round-end sample holder of diameter 13 mm. The measurements were done at the outer tensile-stressed side of the BL-FC sample, while the inner side was exposed to the planar compressive stress. MOKE surface-sensitive hysteresis loops with the magnetic field applied along the ribbon axis (Figure 4(a)) as well as the changes of domain patterns (Figure 4(b)) in comparison to the nonstressed samples clearly confirm the origin of easy and hard magnetization axis on the air and wheel side due to coiling. Different magnetic behaviour observed at both sides is connected, however, with nonidentical size and sign of magnetostriction coefficients (see Table 1). Similarly, positive sign of *λ*_*s*_ in both layers of BL-FF ribbon induces the easy magnetization axis in the ribbon axis at both surfaces.

**Figure 5:**
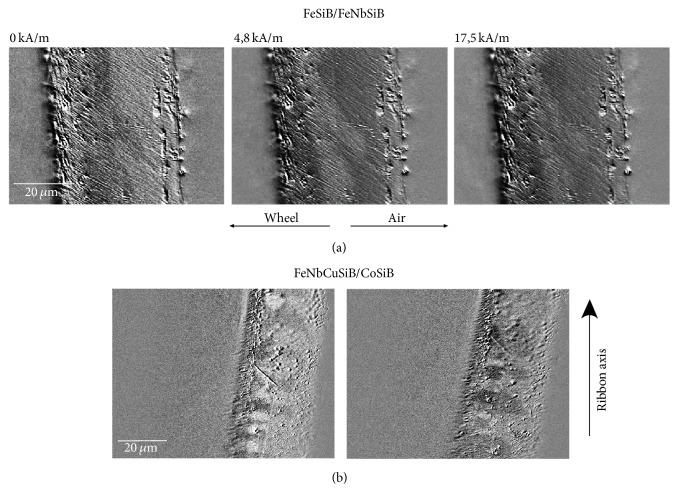
MOKM magnetic domain patterns observed at cross section of BL-FF (a) and BL-FC (b) ribbon. The dependence on applied magnetic field is shown for BL-FF sample, while the domains obtained from two places in remnant state are presented for BL-FC sample.

The magnetic behaviour observed at the ribbon interface using the MOKM is shown in Figure 5. Movement of domain walls in the BL-FF sample is presented at Figure 5(a), while magnetic domains observed in remnant state of BL-FC ribbon are shown in Figure 5(b). The patterns were obtained in the longitudinal configuration, where the plane of light incidence is parallel with magnetic field applied along the interface between layers. As was already discussed in the previous papers [13, 28] the behaviour of magnetic domains is influenced mainly by the magnetostriction of individual layers. Both layers of BL-FF sample have positive magnetostriction coefficients and domains propagate separately in each of them. The domains move as whole blocks from surface towards the interface (or vice versa) with walls parallel to the ribbon interface. BL-FC sample consists of the layers with magnetostriction of opposite signs and exhibits higher tendency to coiling. Typical domain patterns similar to the “chess-board” with alternating black and white (grey) fields are detected. Domains are separated by the walls that are perpendicular to the interface inside the layer and touch the interface on the boundary between layers avoiding the domain propagation in the second layer. Movement of domains along the interface is observed contrary to the BL-FF sample.

### 3.2. Magnetic Force Microscopy

Due to higher roughness of the ribbon wheel sides (see Table 1) only the air sides of BL-FF (FeSiB side) and BL-FC (FeNbCuSiB side) samples have been investigated by AFM/MFM.

**Figure 6:**
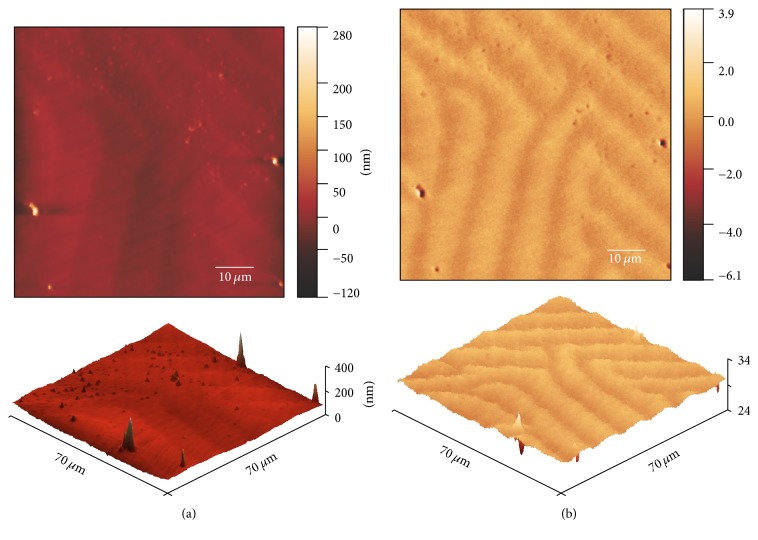
The surface topographies (AFM (a)) and their corresponding magnetic images (phase shift, MFM (b)) observed from the air side of FeSiB/FeNbSiB (BL-FF) sample. 3D images are presented at lower subplots. Apart from a clear magnetic domains structure, a few artefacts due to protrusions on the surface structure comparable to the lift of the tip in the MFM second pass (250 nm) can be seen in magnetic images.

**Figure 7:**
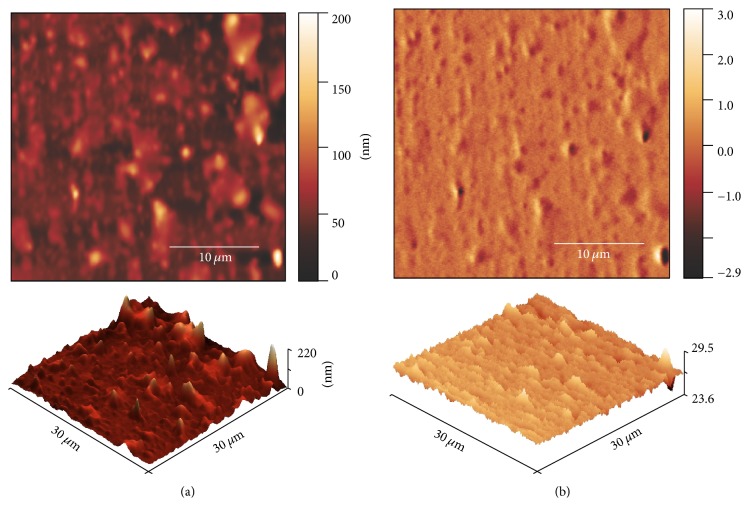
The surface topographies (AFM (a)) and corresponding magnetic images (phase shift, MFM (b)) observed from the air side of FeSiNbCuB/CoSiB (BL-FC) sample.

Figures 6 and 7 show the experiments without the presence of external magnetic field, where (a) corresponds to the AFM topography and (b) corresponds to the magnetic image (phase shift, MFM). The BL-FF structure (Figure 6) consists of crossed stripe domains. The orientation of magnetization in bright and/or murky regions is very similar to the fingerprint ones measured by MOKM, indicating the local perpendicular anisotropy. Sensitivity of MFM to out-of-plane magnetization components has been published by many authors analysing the properties of magnetic materials [17, 21, 26, 27].

**Figure 8:**
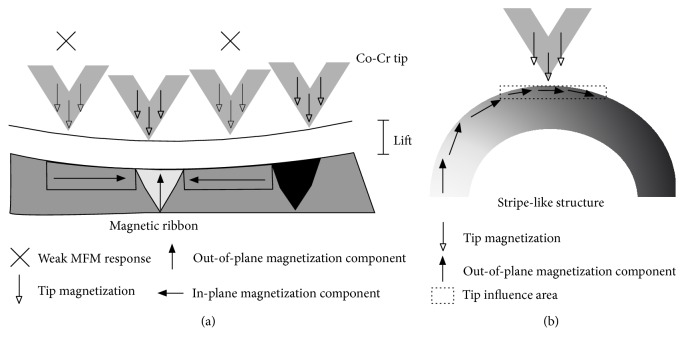
(a) Sensitivity of MFM to the in-plane and out-of-plane magnetization components at the surface of bilayered ribbons. (b) Tip influence area in the case of cross stripe structure.

However, there are also places, where the crossed stripe domains completely vanish. Those regions can be found on the surfaces of both ribbons. An example of such situation is depicted in the case of BL-FC sample (Figure 7). We expect that in these places the wide curved domains observed by the MOKM are present, but they have not been detected by MFM probably due to their large size (see MOKM experiments) and low sensitivity of MFM to locally homogeneous magnetic field and in-plane magnetization components. Both mentioned cases on the surface of amorphous ribbons are schematically sketched in Figure 8(a). Nevertheless, the situation presented in Figure 7 is not quite clear. Although MFM image is closely related to the topography, it is unlikely that the observed fluctuations are just topography artefacts, as the second pass MFM lift height was high above the surface structures (250 nm) and the influence of the topography should be suppressed.

Figure 8(b) schematically explains low MFM contrast of observed cross stripe domains. It is generally known that, to minimize the magnetoelastic anisotropy energy of the system, the closure domains are formed in small areas close to the surface. Therefore, the out-of-plane domains pass continuously into the in-plane domains in the near-surface region and the whole pattern looks like a horseshoe. For cross stripe structure the horseshoe is very broad and the tip influence area reflects only its apex. Therefore one can see the surface closure domains having greater in-plane and weaker out-of-plane magnetization components. MFM phase contrast is then much weaker due to low interaction between the tip and the surface. These results are in good agreement with MOKM detection of fingerprint domains.

**Figure 9:**
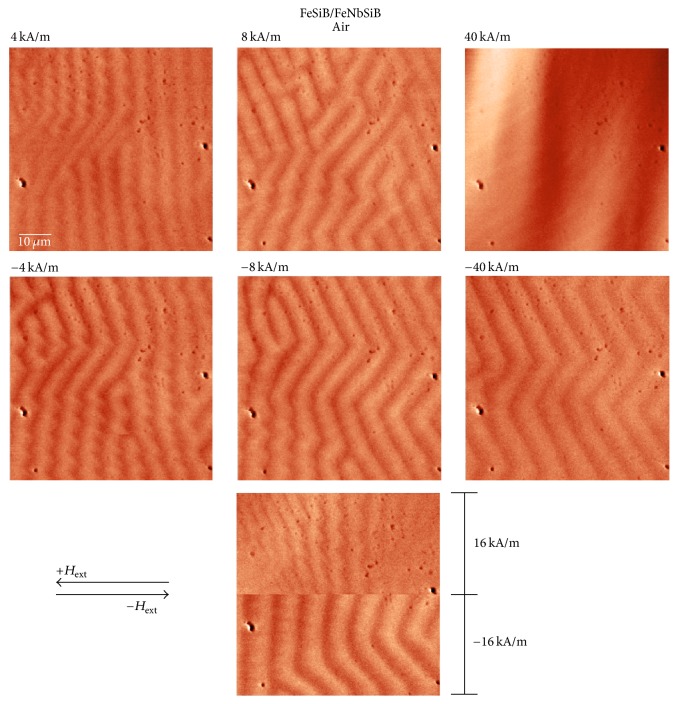
Selected MFM images of BL-FF surface with applied external magnetic field *H*_ext_. The direction of *H*_ext_ is indicated by the arrows. The bottom figure is divided into two parts each corresponding to the positive and negative values of applied field during the same measurement, respectively.

In Figure 6 the BL-FF ribbon was investigated without the presence of external magnetic field. The same place was used for analysis of MFM domains with an applied in-plane external magnetic field *H*_ext_. Results are shown in Figure 9. The direction of *H*_ext_ is indicated by the arrows. The bottom figure is divided into two parts each corresponding to the positive and negative values of applied field during the same measurement, respectively, where upper and lower row of images refer to the domain structure obtained for positive and negative values of *H*_ext_ = 4, 8, 16, and 40 kA/m. Because both the sample and the tip are exposed to the in-plane external magnetic field, their out-of-plane magnetization components become weaker at the expense of increasing the in-plane ones. This influences the magnetic interaction between the tip and the sample and stripe domain patterns differ from that observed without applied *H*_ext_. One can see slow broadening of the stripes with increasing *H*_ext_. At 16 kA/m the sample is partly saturated and the magnetic domain structure completely disappeared after reaching 40 kA/m. Then the external magnetic field was switched off (remnant state) and its amplitude was further increased with the opposite polarity. However, the remnant magnetization of the sample from the previous step is responsible for changes in the domain patterns in comparison to the positive magnetic field polarity. Therefore, stripe domain arrangement is visualized even at 40 kA/m showing practically symmetric zig-zag domain pattern.

## 4. Conclusions

Combination of magneto-optical Kerr microscopy (MOKM) and magnetic force microscopy (MFM) was successfully used for observations of magnetic domain patterns at surfaces and at cross section of bilayered FeSiB/FeNbSiB and FeNbCuSiB/CoSiB ribbons. The following types of domains have been detected and discussed.

*Fingerprint Domains*. Surface closure domains indicating the presence of out-of-plane magnetic anisotropy in the ribbon bulk, coming from local planar compressive stresses originated during the ribbon preparation. MOKM shows high surface magnetic contrast arising from strong in-plane magnetization component and weak out-of-plane magnetization component; MFM exhibits lower magnetic contrast due to the sensitivity to weak out-of-plane magnetization component. MFM response completely vanishes in external magnetic field of 40 kA/m due to the ribbon saturation.

*Wide Curved Domains*. In-plane magnetic domains coming from local tensile stresses, their directions reflect random orientation of local in-plane easy magnetization axis on the surface. MOKM detects wide curved domains at the outer tensile-stressed side thanks to the sensitivity to in-plane magnetization component; practically no corresponding MFM response was observed.

## Figures and Tables

**Table 1:** Basic structural and magnetic characteristics of the FeSiB/FeNbSiB and FeNbCuSiB/CoSiB samples: *H*_*c*_: coercive field, *M*_*s*_: saturation magnetization, *λ*_*s*_: magnetostriction coefficient, *R*_a_: arithmetic average height, *d*: average interface thickness, *T*_*c*_: Curie temperature, and *T*_cryst_: crystallization temperature.

	Side	*H*_*c*_ [A/m]	*M*_*s*_ [Am^2^/kg]	*λ*_*s*_ [ppm]	*R*_*a*_ [nm]	*d* [*µ*m]	*T*_*c*_ [K]	*T*_cryst_ [K]
FeSiB/FeNbSiB	air/wheel	19.2	166.2	+32/+11.8	4/161	1–5	720	750
FeNbCuSiB/CoSiB	air/wheel	18.4	104.7	+18/−2.6	13/47	1–5	700	800

**Table 2:** Basic parameters of the cantilever.

Cantilever length (*µ*m)	Cantilever width (*µ*m)	Cantilever thickness (*µ*m)	Resonance frequency (kHz)	Force constant (N/m)
225	32	2.5	50–70	1–1.6

Curvature radius: 40 nm; magnetic coating: CoCr (thickness about 40 nm).
